# Characterization of Mixed *x*WO_3_(1-*x*)Y_2_O_3_ Nanoparticle Thick Film for Gas Sensing Application

**DOI:** 10.3390/s100505074

**Published:** 2010-05-25

**Authors:** M. H. Shahrokh Abadi, M. N. Hamidon, Abdul Halim Shaari, Norhafizah Abdullah, Norhisam Misron, Rahman Wagiran

**Affiliations:** 1 Electrical and Electronic Department, Engineering Faculty, Universiti Putra Malaysia 43400, Serdang, Selangor, Malaysia; E-Mails: mnh@eng.upm.edu.my (M.N.H.); norhisam@eng.upm.edu.my (N.M.); rwagiran@eng.upm.edu.my (R.W.); 2 Physics Department, Science Faculty, Universiti Putra Malaysia 43400, Serdang, Selangor, Malaysia; E-Mail: ahalim@fsas.upm.edu.my; 3 Department of Chemical and Environmental of Engineering Faculty, Universiti Putra Malaysia 43400, Serdang, Selangor, Malaysia; E-Mail: fizah@eng.upm.edu.my

**Keywords:** gas sensor, thick film, nano powder, tungsten trioxide, yttrium oxide

## Abstract

Microstructural, topology, inner morphology, and gas-sensitivity of mixed *x*WO_3_(1-*x*)Y_2_O_3_ nanoparticles (*x* = 1, 0.95, 0.9, 0.85, 0.8) thick-film semiconductor gas sensors were studied. The surface topography and inner morphological properties of the mixed powder and sensing film were characterized with X-ray diffraction (XRD), atomic force microscopy (AFM), transmission electron microscopy (TEM), and scanning electron microscopy (SEM). Also, gas sensitivity properties of the printed films were evaluated in the presence of methane (CH_4_) and butane (C_4_H_10_) at up to 500 °C operating temperature of the sensor. The results show that the doping agent can modify some structural properties and gas sensitivity of the mixed powder.

## 1. Introduction

Adsorption of a gas on the surface of a metal oxide semiconductor material can bring about a significant change in the electrical resistance of the material. During the conversion, a number of physical and chemical parameters such as film thickness, grain size, intergrain contact, porosity, grain network, phase composition, elemental composition, bulk stoichiometry, surface architecture, type of additives and dopants, *etc.* are involved in the changes of conductance of the oxide when the film is exposed to a gaseous atmosphere [1–8]. Among these parameters, growth and adding dopants and additives into the oxide strongly affects the surface properties and structure of the metal oxide gas sensors. Oxides such as SnO_2_, WO_3_, and ZnO [9–12] have been considered by many researchers in the field of semiconductor gas sensors. Although pure oxides, individually, are sensitive to a range of gases, they also have their own detection issues such as cross-sensitivity, sensitivity to humidity, shorter life time, lack of ability to detect a certain gas, higher temperature of reaction and so on.

Some additives such as noble transition metals do not participate in the reaction phase, but promote the improvement of sensitivity of the sensor to be strongly sensitive to a certain type of gas, decrease response and recovery times, improve thermal stability of the overall structure and sensor properties, and modify the catalytic reactivity and morphology of deposited films [13–17]. Small quantities of dopants in oxide forms such as TiO_2_ [18], Bi_2_O_3_ [19], MoO_3_ [20], NiO [21], *etc*. modify the microstructure, suppress the grain growth, and enhance the porosity of the basic oxide, leading to an increase of film sensitivity toward a certain gas, and reducing the reaction temperature to as low as room temperature [22]. Besides, dopants can promote the speed of reaction in presence of certain gas *versus* a longer response and recovery time for some other gases [18,23]. They also can improve sensitivity of the sensor to humidity and eliminate cross-sensitivity [24,25].

In previous works, sensitivity of a planar resistive gas sensor based-on WO_3_:Y_2_O_3_ were studied [1,2]. It is well known that the resistance of WO_3_ film as an *n*-type semiconductor material is increased in the presence of oxidizing gases and decreased when exposed to reducing gases [11,26]. Besides, yttrium oxide, as an n-type semiconductor material, may exhibit dehydration or dehydrogenation properties, depending on its pretreatment, in decomposition reactions of alcohols; it may also be a catalyst for the hydrogenation of olefins [27]. Therefore, the combination of these two oxides is supposed to produce an *n^+^*-type semiconductor material and the final product would be more sensitive to some gases, and the humidifying dependency of the mixed films should probably decrease due to the presence of yttria. In this paper, microstructural, morphology, and gas sensitivity of WO_3_ doped with Y_2_O_3_ in the presence of methane (CH_4_) and butane (C_4_H_10_) is studied.

## 2. Details of Experimentation

A series of *x*WO_3_(*1-x*)Y_2_O_3_ (*x* = 1, 0.95, 0.9, 0.85, 0.8) samples was prepared by ultrasonically mixing and ball milling of two primary powders: WO_3_ (Aldrich >99.9%) and Y_2_O_3_ (Aldrich >99.9%). Mixing was performed inside an Erlenmeyer flask in an ultrasonic bath (Grant Instrument XB2) for 24 hours while m-Xylene was used as medium. Milling was performed using different type of ceramic balls (*d*: 4 to 11 mm) and ceramic cylinders (*d* = 10 mm, *l* = 10 mm) in presence of m-Xylene for 48 hours. Samples then were dried at 55 °C in an oven for 1 hour and sintered at 950 °C for 6 hours.

After sintering, samples were mixed with 5 *wt.%* glass binder contained SiO_2_, B_2_O_3_, Al_2_O_3_, and Bi_2_O_3_ and ground in an agate mortar while drops of organic vehicle was adding to make a homogenous viscose paste.

**Figure 1.**
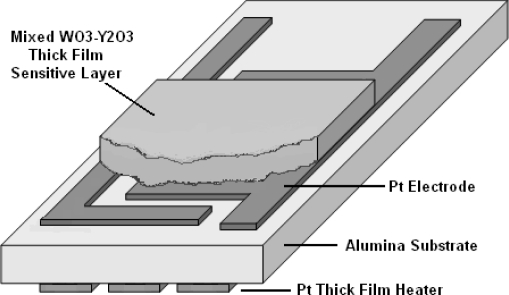
Configuration of the sensor layers.

Sensors were prepared and fabricated by printing and firing paste like thick film on an interdigitated platinum electrode provided on one side of an alumina substrate (99.6%, 0.25 mm thick) with a platinum meander shape as heater on the back side to heat up the film at desired temperatures. A polyester screen with an emulsion layer of 5 ± 2 μm thick was chosen and printing was performed in printing screen machine (DeK J1202RS). First the heater layer was printed and dried at 150 °C inside a belt furnace (DeK J HD-450), then the electrode layer was printed and dried at same temperature for 10 minutes and subsequently it was fired at 980 °C for 10 minutes. Afterwards, the sensing layer was printed in an area of 6 × 5 mm^2^ onto the electrode layer and firing temperature was kept at 650 °C for 10 minutes with an ascent and descent range of 10 °C/min because annealing temperature above 700 °C will destroy the sensing layer due to an intensive chemical reaction between sensing layer with the electrodes and substrate [11]. Figure 1 shows the structure of the sensor layers.

The structure of films was analyzed by X-ray diffraction (X-Ray Diffractometer X’PERT PRO PW3050) performed with Cu radiation as anode material between *2θ* = 10.017° and 79.944° (Step Size *2θ* = 0.033°, Scan Step Time = 19.685 s, Operating Voltage = 40 kV, Operating Current = 30 mA, Divergence Slit Size = 0.0286°, and *λ* = 1.542 Å). Microstructure of the films was also determined by SEM (HITACHI S-3400) at an emission current between 60 μA to 68 μA and acceleration voltage of 15.0 kV while working distance was set at 4.8 mm. Before the SEM analysis, sensing area of the films was coated with 35 nm thick gold. The surface morphology of the sensitive films was also investigated by atomic force microscopy (AFM, SPA300HV, 2 channel simulated mode, deflection type).

Samples were exposed to different concentrations of methane and iso-butane as target gases under controlled condition of humidity and temperature ambient inside a 6860 cc test chamber. Nitrogen was used to purge the chamber before exposure, in which the ambient humidity and temperature inside the chamber were kept at 55% ± 2% and 28 °C ± 3 °C, respectively. A 100 μL Hamilton syringe was used for applying the target gases, with the nitrogen cylinder connected to the chamber via a control valve directly. The conductivity of the films was derived from the measurements of the voltage across a load resistor in series with the sensor resistor (*R_s_*) using a data logger (Pico Technology ADC-20) and temperature dependency of the film conductivity *versus* gas concentration was measured in the temperature range of 100–500 °C.

## 3. Results and Discussion

### 3.1. Structural Analysis of Powders

#### 3.1.1. X-ray Diffraction Studies

**Figure 2.**
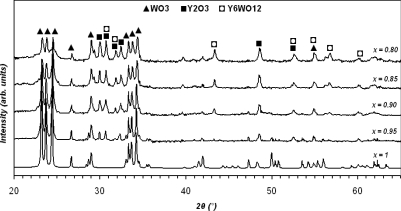
XRD analysis of mixed powder for *x* = 1, 0.95, 0.9, 0.85, and 0.8.

Figure 2 shows the XRD patterns of mixed *x*WO_3_(*1-x*)Y_2_O_3_ powders for 2*θ* = 20°∼65°. The main peaks for all samples are the same as the *x* is changed (matched with 1-0486, 2-0308, 5-0363, 44-0357, and 44-0399 JCPDS file numbers). A higher level of doping (*x* ≤ 0.85) shows a significant change in chemical composition as Y_6_WO_12_ is produced. Furthermore, the distance between similar atomic planes (d-spacing) in WO_3_ and Y_2_O_3_ is changed with Δ*d* variation less than 10% as *x* is decreased. Maximum d-spacing of each sample occurs at a mean peak of XRD pattern and also can be determined from the Bragg Equation (*nλ* = 2*d*sin*θ* for *n* = 2, Bragg angel = *θ*, and *λ* = 1.542 Å). In the case of *x* = 1 and at *θ* = 23.184°, d-spacing from the XRD result is 3.8293 Å, whilst calculated d-spacing from the Bragg Equation is 3.9168Å.

The average of crystallite size was determined from XRD results based-on the Scherrer Equation (Scherrer Constant, *K* = 0.94 for spherical crystals with cubic symmetry [28] and *λ* = 1.542 Å from XRD results):

(1.a) $$\mathrm{FWHM}=\frac{K\lambda }{L\;\text{cos}\theta }$$

then:

(1.b) $$L=\frac{K\lambda }{(\mathrm{FWHM})\;\text{cos}\theta }$$

**Figure 3.**
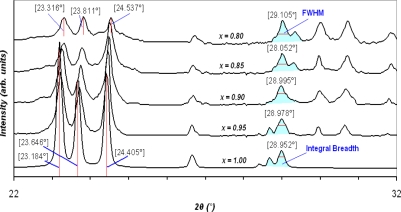
A sample of shift in peak position and FWHM variations in XRD pattern.

**Figure 4.**
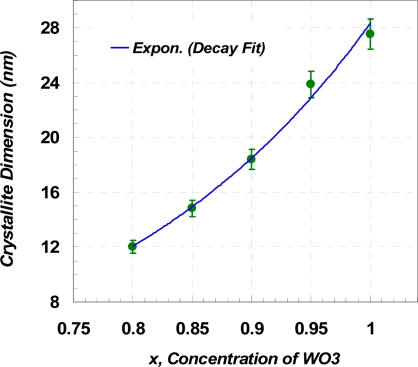
Average crystallite sizes of *x*WO_3_(*1-x*)Y_2_O_3_ determined by the Scherrer Equation.

Since *K* and *λ* are constant, the crystallite size is only related to the position (2*θ*) and the Full-Width Half-Maximum (FWHM) value of the peaks. Table 1 shows the maximum, minimum, and average crystallite sizes of the samples determined from Equation (1.b). Note that the calculation was carried out for the first 12 peaks of the XRD results. Variations into the crystallite sizes in respect to Y_2_O_3_ concentration can be interpreted as the changes into FWHM values and a shift into position angel of the peaks. Figure 3 shows that the slope of the XRD patterns as well as peak positions are changed due to the level of dopant. Increasing the slope leads to a higher value of integral breadth, this in return results in an increased FWHM value. Decrease of *x*, resulted in slightly displacement of the peak position, 2*θ*, to higher values, which in return causes a reduction of cos*θ*. However, this reduction is not significantly changed the value of crystallite size, *L*, in the equation (1.b). Furthermore, the value of shift in the position of peaks is not unique for the whole pattern and varies from one peak to another. The maximum displacement of the peak position observed, with respect to the basic powder, happened at the fourth highest peak of WO_3_ from 2*θ* = 34.074° (*x* = 1.0) to 2*θ* = 34.371° (*x* = 0.80). The plot of crystallite dimension *versus* molar content of WO_3_ is depicted in Figure 4. The line shows that the sizes are almost fitted with the exponential decay curve.

#### 3.1.2. Transmission Electron Microscopy (TEM) Studies

**Figure 5.**
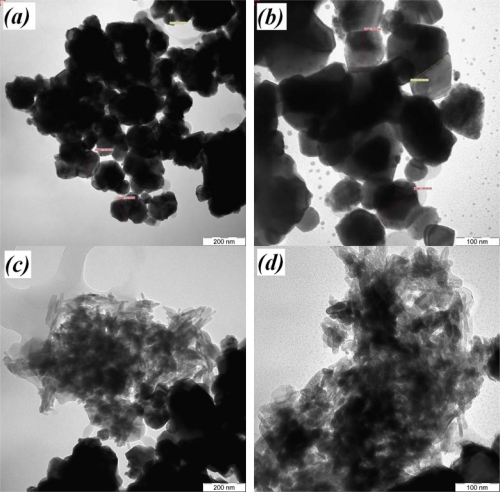
TEM micrographs of *x*WO_3_(*1-x*)Y_2_O_3_: (a) *x* = 1.0, (b) *x* = 0.95, (c) *x* = 0.90, (d) *x* = 0.80.

Figure 5 shows TEM micrographs of the powders obtained from pure WO_3_, and 5 *wt.%*, 10 *wt.%*, and 20 *wt.%* Y_2_O_3_ doped into WO_3_. TEM images show groups of nanosized particles ranging below 85 nm (mean particle size, 63 nm). The sizes of particles were larger than the crystallite sizes measured by XRD because the scattering measures the average lengths between the defects. Spherical crystallites in undoped samples (Figure 5a) and 5 *wt.%* doped (Figure 5b) have sizes ranging from 22 nm to 85 nm and almost show an angular hexagon. At higher levels of doping (Figure 5c and 5d), the aggregates of particles become elongated and rod-shape with an average lengthened and broadness of 65 nm and 15 nm, respectively. However, reduction of particle sizes due to the dopant concentration has been reported by the other researchers [10,20,29].

### 3.2. Microstructural Analysis of Sensitive Films

#### 3.2.1. Atomic Force Microscopy (AFM) Studies

**Figure 6.**
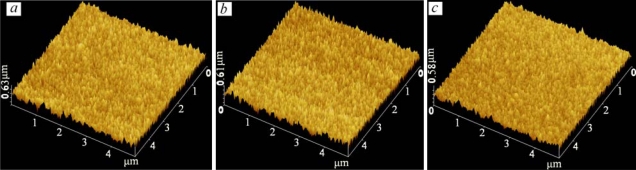
AFM micrograph of a sensing area of 5 × 5 μm^2^ for (a) *x* = 1, (b) *x* = 0.90, and (c) *x* = 0.80.

The surface microstructure changes of films that were about 15 μm thick over a 5 μm × 5 μm area due to the level of doping were studied. AFM micrographs of the undoped, and 10 *wt.%* and 20 *wt.%* Y_2_O_3_ doped into WO_3_ films are shown in Figure 6. Analysis was run under two channel simulated mode, channel A for topology (Z sensing) and channel B for deflection. The films show an extremely smooth surface with a roughness of about 0.6 μm. From grain size analysis of AFM and for the given area of the films, the average size of 3.324E + 4 nm^2^ ± 5% and average diameter of 200 ± 35 nm were observed. The AFM micrographs confirmed that the Y_2_O_3_ concentration was not an important factor for the porosity and roughness of the sensing area.

#### 3.2.2. Scanning Electron Microscopy (SEM) studies

**Figure 7.**
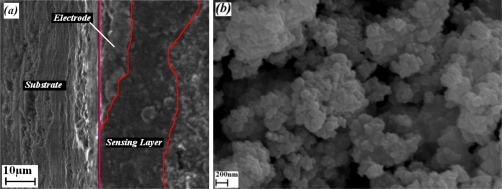
SEM micrographs: (a) cross-section of sensor layer, (b) surface of sensing film.

Figure 7a shows a SEM micrograph of the cross section film layer deposited by screen printing. A 10 μm thick Pt electrode and a 15 μm thick sensing layer can be observed. The surface of sensing area in Figure 7b shows porosity as the typical microstructure of screen printed sensing films. The surface porosity observed was not a ruling factor of the doping concentration as it was also demonstrated in AFM results. The porosity of the sensing layer definitely resulted from the annealing temperature, such that the films annealed at lower temperature exhibit a compact flat surface with no crystalline structure, and for film annealed at higher temperature, the surface becomes rough and granules appear, and the average grain size increases, while the film becomes more porous [11,20,30]. Therefore, the porosity doesn’t change since the annealing temperature in this experiment was kept at 650 °C. The average grain size measured in the SEM images is very close to those obtained from AFM results.

### 3.3. Electrical Characterization

#### 3.3.1. Effect of Doping on Electric Resistance

**Figure 8.**
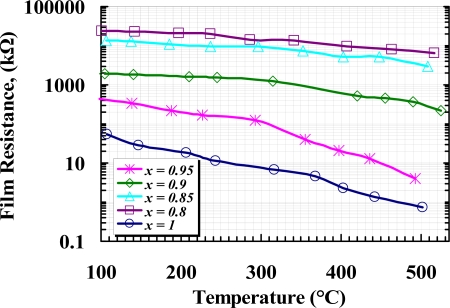
Sensor resistance *versus* sensor temperature for all samples.

Electric resistance of the samples was measured at various temperatures ranging from 100 °C to 525 °C before applying gas. Figure 8 shows the variation of film resistance *versus* temperature for all samples, where the resistances converted to the logarithm was plotted against temperature for the given range. The results show the resistance of all the samples decreased with increasing temperature within the given temperature range. A magnificent increase in the resistance of films can be observed due to the concentration level of Y_2_O_3_. The increase of resistance due to the molar concentration of yttria can be interpreted as an increasing in the depletion layer thickness. Since some site of *W* is replaced with *Y*, as it was illustrated in XRD results, the majority of carrier concentration in *W* is decreased, resulting in an increase in Schottky barrier height at the grain boundaries. The minimum resistance observed at *T* = 502 °C was 0.723 kΩ for undoped WO_3_, while it was 6,456 kΩ for 20 *wt.%* added Y_2_O_3_ at *T* = 508 °C.

#### 3.3.2. CH_4_ Sensing Properties

**Figure 9.**
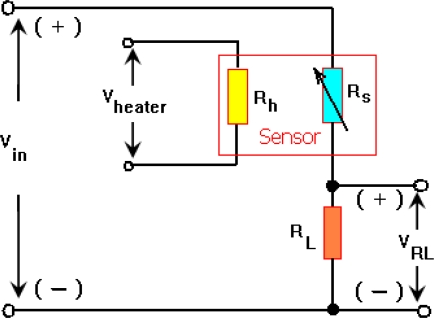
Setup experiment for measuring sensor response.

Figure 9 shows the setup experiment for measuring response of the samples. A dc-voltage is applied across the heater (*V_h_*) and is changed corresponding to the sensor temperature. A fixed dc voltage is also applied to the *V_in_* and voltage across the *R_L_* (*V_RL_*) is measured. The maximum value of *V_RL_* is located at minimum value of sensor resistance, *R_S_*. The load resistance, *R_L_*, is chosen to prevent the sensor from saturation. Then *R_S_* as well as sensitivity of sensor (*S*) is determined from the Equations (2) and (3), respectively:

(2) $$R_{S}=\frac{V_{\mathrm{in}}-V_{\mathrm{RL}}}{V_{\mathrm{RL}}}\times R_{L}$$

(3) $$S=\frac{R_{S,a}}{R_{S,g}}$$

where *R_S,a_* is the resistance of sensor in air and *R_S,g_* is the resistance of sensor in presence of gas.

**Figure 10.**
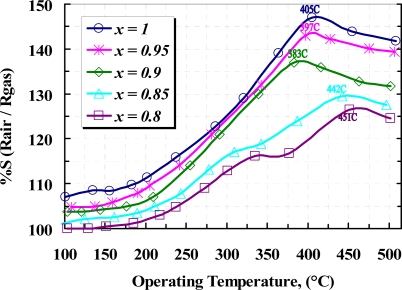
Sensitivity of *x*WO_3_(*1-x*)Y_2_O_3_ sensor to 1,000 ppm methane *versus* operating temperature.

The sensitivity prosperities of samples in the presence of methane were tested inside a test chamber and nitrogen was used for purging. It is well known that the sensitivity of a gas sensor is extremely dependent on the operating temperature of the sensor as well as the concentration of doping [14–20]. Such dependencies for detection of 1,000 ppm CH_4_ are depicted in Figure 10. From this figure, the maximum sensitivity of the samples is located at about 405 °C for pure film. It can be seen that the concentration of yttria up to 10 *wt.%* shifts the maximum sensitivity to a lower operating temperature, but maximum sensing in samples with more than 10 *wt.%* yttria content take place at operating temperature above 405 °C. Those shifts can be explained as follow:

The gas absorbance mechanism of WO_3_, as an *n*-type semiconductor material, is obviously dependant on the amount of chemisorbed oxygen ions on the surface [11]. The chemisorption of a reducing gas such as CH_4_, needs the electrons from the surface of the film which is resulted in a change in the surface charge (e.g., OH^−^→O^2−^, O^−^→O^2−^, or O^2−^→2O^−^). In the case of complete reaction, methane molecules react with oxygen ions trapped between grains boundaries caused to increase charge carriers and resulted to decrease film resistance:

(4) $${\text{CH}}_{4}+4O^{-}\to {\text{CO}}_{2}+2H_{2}O+4e^{-}$$

Furthermore, increasing concentration of Y_2_O_3_ caused a change in the activation energy (*E_a_*) and slop of Schottky barrier (*Δφ*) over the operating temperature range. Since the conductivity of the film is related to the activation energy, height of Schottky barrier, and operating temperature (*σ*∼exp(–*eΔφ/kT*), *σ*∼exp(*E_a_/kT*)); any changes either in operating temperature or carrier concentration would result in changes in the film conductivity.

**Figure 11.**
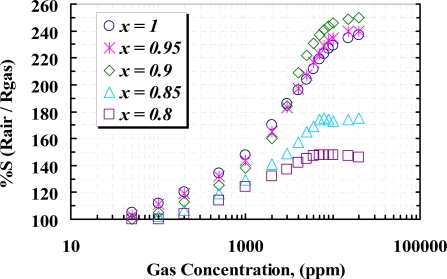
Sensitivity of samples to different concentration of CH_4_ at operating temperature of 405 °C (*x* = 1), 395 °C (*x* = 0.95), 380 °C (*x* = 0.90), 440 °C (*x* = 0.85), and 450 °C (*x* = 0.80).

Figure 11 shows the responses of the samples *versus* different concentrations of CH_4_ (50 ppm ∼ 20,000 ppm), measured at the temperature corresponding to the maximum response to 1,000 ppm CH_4_. It can be seen that the responses rapidly increase due to increasing CH_4_ concentration up to about 1% (10,000 ppm) and then a slightly change in the response at higher concentration of methane can be observed. Undoped (*x* =1) sensor shows greater response than the doped samples at about 3,000 ppm and below, but at higher concentration, 5 *wt.%* (*x* = 0.95) and 10 *wt.%* (*x* = 0.90), doped sensors show higher response with respect to the other samples. From the TEM and XRD results, it can be seen that at a concentration of yttria higher than 10 *wt.%*, crystallography of WO_3_ shows the particles are agglomerated and tendto a rod-like structure, due to the contents of yttria and sintering temperature, as the yttria is a well known sintering aid factor [31,32]. Below 10 *wt.%* content, the grain sizes are increased, so that the 10 *wt.%* yttria content can modify the operating temperature as well as the sensor response. At concentrations above 10,000 ppm of methane, the rate of recombination and generation of surface charges would be constant; therefore no more reaction between CH_4_ molecules and surface charges happens and the sensor would be saturated. Concentration of yttria higher than 10 *wt.%* causes a high gradient of barrier potential, which wouldlead to high surface resistivity and lower sensitivity.

**Figure 12.**
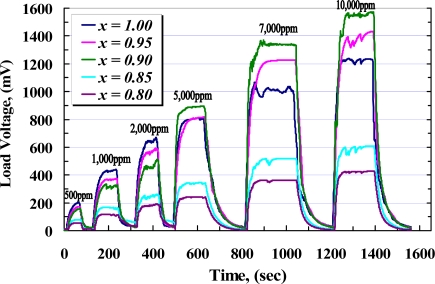
Response of samples to 0.05%, 0.1%, 0.2%, 0.5%, 0.7%, and 1% CH_4_, measured at operating temperatures of 405 °C (*x* = 1), 395 °C (*x* = 0.95), 380 °C (*x* = 0.90), 440 °C (*x* = 0.85), and 450 °C (*x* = 0.80).

While the sensors are exposed to target gas, the sensor resistance decreases rapidly with time to be stable at a value in which the complete reaction happens. On the other hand, the complete reaction takes a place at a certain temperature so that it would be incomplete below and above that temperature, as it was illustrated at Figure 10. The time taken from the exposure until the response is stable is called the response time (*t_r_*), while the taken time throughout removing gas until the response is stable at its minimum value is called the recovery time (*t_rr_*). Figure 12 shows the response of samples during exposure to different concentrations of CH_4_ measured across the load resistor. From this figure and with respect to Figure 9, it can be seen that during exposure, higher concentration of methane causes *V_RL_* increase and voltage across *R_S_* is decreased rapidly with almost the same response time for each sample, while at higher concentration of CH_4_ recovery time is extended.

#### 3.3.3. C_4_H_10_ Sensing Properties

The chemisorption of C_4_H_10_ during a complete reaction with trapped negative ions of oxygen produces water vapor and carbon dioxide. If the operating temperature is not sufficient or the oxygen ions are limited, soot or carbon monoxide may also be formed:

(5) $$2C_{4}H_{10}+13O_{2}^{-}\to 8{\text{CO}}_{2}+10H_{2}O+13e^{-}$$

where the negatively charge carriers, produced during the reaction, cause a fast degradation in the resistance of the sensor.

**Figure 13.**
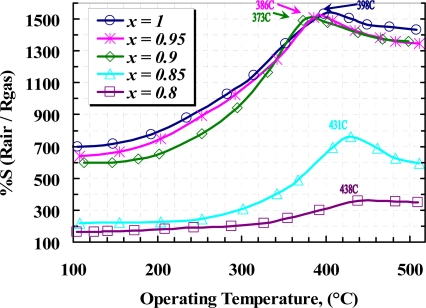
Sensitivity of *x*WO_3_(*1-x*)Y_2_O_3_ sensors to 1,000 ppm iso-butane *versus* operating temperature.

**Figure 14.**
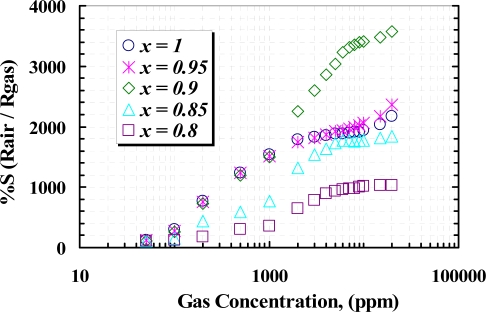
Sensitivity of samples to different concentrations of C_4_H_10_, measured at operating temperatures of 395 °C (*x* = 1), 385 °C (*x* = 0.95), 370 °C (*x* = 0.9), 430 °C (*x* = 0.85), and 440 °C (*x* = 0.80).

The temperature dependency of the reaction and the optimized operating temperature, where an almost complete reaction take place, are investigated in the presence of 1,000 ppm iso-butane in a temperature range between 100 to 500 °C. Figure 13 shows such a relation between the operating temperature and the normalized resistance of the samples at 0.1% of C_4_H_10_ air content, in which the pure film has the higher sensitivity than the others, meanwhile a lower operating temperature was observed in 10 *wt.%* doped film. Figure 14 shows the sensitivity of the samples exposed to a range of C_4_H_10_ at their optimized operating temperature, where the 10 *wt.%* film shows a magnificent sensitivity to above 0.2% of iso-butane content. Furthermore, *x* = 0.9 sample shows an almost linear of sensitivity for whole range of the gas concentration.

**Figure 15.**
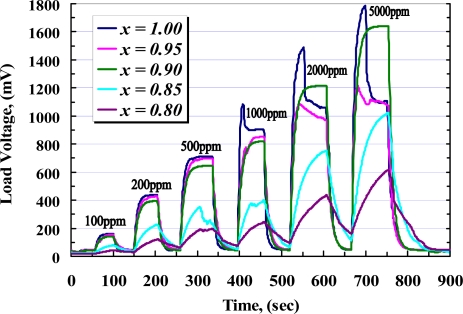
Response of samples to 0.01%, 0.02%, 0.05%, 0.1%, 0.2%, and 0.5% C_4_H_10_, measured at operating temperatures of 395 °C (*x* = 1), 385 °C (*x* = 0.95), 370 °C (*x* = 0.9), 430 °C (*x* = 0.85), and 440 °C (*x* = 0.80).

To investigate the response and recovery time of the samples, C_4_H_10_ was applied in different concentrations from 100 to 5,000 ppm. Figure 15 shows those responses of the samples during exposure to the gas. From this figure and Figure 14, it seems that the responses of pure and 5 *wt.%* doped sample are almost the same for the whole range of the applied gas. When the concentration of iso-butane is increased above 1,000 ppm, an overshooting in response of the pure film is observed, while the response of 10 *wt.%* added yttria shows a higher sensitivity than the other films in the steady state. From Figure 15 it can be seen that the peak of overshooting is increased due to increasing of the gas concentration. Such overshooting in gas concentration is considered to show up as a result of competition between diffusion and reaction for the gas molecules having entered from the surface to the bulk [33]. In consequence of a higher Schottky barrier due to increasing the ratio of dopant, the overshooting is eliminated in a higher concentration of yttria. Also, it can be seen that the films with dopant concentration above 15 *wt.%* show a poor response and recovery times.

## 4. Conclusion

The effect of 5, 10, 15, and 20% nano-powder yttria added into tungsten trioxide was determined. The results show that the crystallite size and d-spacing of WO_3_ can be modified by adding Y_2_O_3_. Furthermore, adding certain concentrations of yttria into WO_3_ moderates the temperature of reaction during detection of the alkanes such as methane and iso-butane. The film containing 10 *wt.%* yttria shows higher sensitivity to those esters especially at concentrations above 2,000 ppm.

## Figures and Tables

**Table 1.** Crystallite size of the samples *versus* level of doping.

***x***	Crystallite size (*nm*)		
	Maximum	Average	Minimum
1.0	29.63	27.53	23.41
0.95	25.47	23.87	19.07
0.90	20.33	18.41	16.94
0.85	17.67	14.82	13.51
0.80	15.37	12.02	11.28
